# A Comparative Study of Mouse Hepatic and Intestinal Gene Expression Profiles under PPAR**α** Knockout by Gene Set Enrichment Analysis

**DOI:** 10.1155/2011/629728

**Published:** 2011-07-27

**Authors:** Kan He, Qishan Wang, Yumei Yang, Minghui Wang, Yuchun Pan

**Affiliations:** ^1^School of Agriculture and Biology, Shanghai Jiao Tong University, Shanghai 200240, China; ^2^Shanghai Key Lab for Veterinary Biotechnology, Shanghai 200240, China

## Abstract

Gene expression profiling of PPAR**α** has been used in several studies, but fewer studies went further to identify the tissue-specific pathways or genes involved in PPAR**α** activation in genome-wide. Here, we employed and applied gene set enrichment analysis to two microarray datasets both PPAR**α** related respectively in mouse liver and intestine. We suggested that the regulatory mechanism of PPAR**α** activation by WY14643 in mouse small intestine is more complicated than in liver due to more involved pathways. Several pathways were cancer-related such as pancreatic cancer and small cell lung cancer, which indicated that PPAR**α** may have an important role in prevention of cancer development. 12 PPAR**α** dependent pathways and 4 PPAR**α** independent pathways were identified highly common in both liver and intestine of mice. Most of them were metabolism related, such as fatty acid metabolism, tryptophan metabolism, pyruvate metabolism with regard to PPAR**α** regulation but gluconeogenesis and propanoate metabolism independent of PPAR**α** regulation. Keratan sulfate biosynthesis, the pathway of regulation of actin cytoskeleton, the pathways associated with prostate cancer and small cell lung cancer were not identified as hepatic PPAR**α** independent but as WY14643 dependent ones in intestinal study. We also provided some novel hepatic tissue-specific marker genes.

## 1. Introduction

Peroxisome proliferator-activated receptor *α* (PPAR*α*) is a ligand-activated transcription factor which is one of members of the nuclear-hormone receptor (NR) superfamily [1–3]. WY14643 is one of synthetic ligands, which is primarily an activator of PPAR*α* [4, 5]. In mammals, high levels of PPAR*α* expression are found in tissues with active fatty acid catabolism, such as liver heart, kidney, brown adipose tissue, muscle, small intestine, and the large intestine [1, 2, 6]. The liver is a central player in the whole body energy homeostasis by its ability to orchestrate fatty acid and glucose metabolism, and it plays an important role in fatty acid oxidation regulated by PPAR*α* [7]. During the past 15 years, numerous studies with PPAR*α* null mice have demonstrated the critical roles played by this receptor in energy metabolism, hepatic steatosis, inflammation, cardiac pathophysiology, cell-cycle alterations, and hepatocarcinogenesis [8, 9]. In recent years, microarray technology has been highly used to map PPAR*α*-dependent genes and further characterizes PPAR*α* function in different tissues in genome wide [10–15]. However, most of gene expression profilings of PPAR*α* has been almost exclusively studied in liver. In liver study, PPAR*α* is shown to be critical for the coordinate transcriptional activation of genes involved in lipid catabolism, including cellular fatty acid uptake and activation, mitochondrial *β*-oxidation, peroxisomal fatty acid oxidation, 

ketone body synthesis, fatty acid elongation and desaturation, and apolipoprotein synthesis [16, 17]. 

In addition, several studies on the functions of PPAR*α* and PPAR*α* target genes in mouse nonhepatic tissues (such as small intestine) had also been done [12, 14, 18]. For example, a genome-wide microarray method was performed to examine the effects of PPAR*α* agonists on the expression levels of all the nutrient/drug plasma-membrane transporters in the mouse small intestine as a result, which show expression levels of seven nutrient/drug transporters such as Abcd3 and Octn2 in the intestine were upregulated and the expression level of one (Mrp1/Abcc1) was downregulated by PPAR*α* [12]. Actually, we could suppose that the regulatory mechanisms of PPAR*α* in mouse tissues are diverse in some cases. Therefore, a comparative study using the same analysis method on genome wide expression datasets between in hepatic and in nonhepatic tissue of mice is essential for us to identify tissue-specific pathways or genes involved in PPAR*α* activation. It may be helpful for us to get comprehensive knowledge on the biological functions of PPAR*α* in distinct tissues of mice. Moreover, in order to evaluate PPAR-dependent and independent effects on tissue-specific gene expression, a null mutation in the PPAR gene was generally used, similarly, with the treatment of WY14643 or not was usually employed to define the role of WY14643 in PPAR activation [13, 19, 20]. Thus, identification of PPAR-dependent or independent pathways and WY14643 PPAR-dependent or independent pathways by comparison of gene expression profiles in different tissues may provide insight into the PPAR-mediated homeostasis.

Nowadays, the analysis of genomic data has become an important challenge in bioinformatics. Currently, the most well-known method named gene set enrichment Analysis (GSEA) has been widely used to analyze gene expression profiles, especially to identify predefined gene sets which exhibited significant differences in expression between samples from control and treated [21, 22]. The algorithms calculate the statistical significance of the expression changes across groups or pathways rather than individual gene, thus allowing identification of groups or pathways most strongly affected by the observed expression changes. The analysis based on a group of relevant genes instead of on an individual gene increases the likelihood for investigators to identify the critical functional processes under the biological phenomena in each study. The goal of GSEA is to determine other interesting categories (pathways), where the constituent genes show coordinated changes in expression over the experimental conditions, other than in the form of sets of differentially expressed genes. Compared to other methods, one of the advantages of GSEA is the relative robustness to noise and outliers in the data, especially for considering the comparison of two different groups, or phenotypes. In other words, it is able to highlight genes weakly connected to the phenotype through pathway analysis which may be difficult to detect by using classical univariate statistics. Moreover, GSEA is likely to be more powerful than conventional single-gene methods in the study of complex diseases in which many genes make subtle contributions. For example, a previous research study has successfully applied GSEA to evaluate the psoriasis transcriptome across different studies, overcoming the shortcomings of overlapping individual differentially expressed genes (DEG) approach [23]. In order to further investigate the pathogenesis of endometriosis, our group have also performed a cross-study GSEA in endometriosis. As a result, we increased the concordance to identify many biological mechanisms involved in endometriosis, which are novel in terms of their connection to endometriosis [24].

Here, we employed two published microarray datasets both PPAR*α* related, respectively, in mouse liver and intestine, and applied GSEA to them in order to compare the regulatory mechanisms of gene expression by PPAR*α* activation between in small intestine and in liver of mice and indentify PPAR*α*-dependent or independent pathways under WY14643 treatment or not. Concretely, PPAR*α*-dependent pathways would be identified by comparing PPAR*α* knockout (KO) to wild type (WT) animals alone, while WY14643 PPAR*α*-dependent pathways would be identified by comparing KO to WT treated with WY14643; WY14643-dependent pathways would be identified by comparing with the treatment of WY14643 or not in WT animal, while PPAR*α*-independent pathways would be identified by comparing with the treatment of WY14643 or not in KO animal.

## 2. Materials and Methods

### 2.1. Microarray Datasets Collection and Preprocessing

We searched GEO (http://www.ncbi.nlm.nih.gov/geo/) for the gene expression profiling studies related to PPAR*α* knockout. Data were included in our reanalysis if they met the following conditions: (1) the data is in genome-wide, (2) comparison was conducted between PPAR*α* knockout and wild type, and (3) complete microarray raw or normalized data are available. Finally, we chose the datasets GSE5475 and GSE8295 for our reanalysis studies, which were both contributed by Guido Hooiveld in different periods, respectively, for mouse small intestine and liver [14, 15]. The summary of these two datasets was shown in Table 1. In those two datasets, pure bred wild type (129S1/SvImJ) and PPAR*α*-null (129S4/SvJae) mice were both treated with the synthetic PPAR*α* ligand WY14643 (0.1% w/w) mixed in the food, or normal food (control) for 5 days. In dataset GSE5475, the complete intestines were then removed and total RNA was isolated, RNA of 3 biological replicates was hybridized to Affymetrix 430A arrays. In dataset GSE8295, liver total RNA from 4 biological replicates was hybridized onto Affymetrix mouse genome 430 2.0 GeneChip arrays. The details of the information about our divided eight studies from these two datasets can be seen in Table 2. On the one hand, we were focused on studying the effects of PPAR*α*-dependent side. Among them, there were four studies. Study 1: Intestine-CT (KO/WT) is the study of mouse small intestine of wild type versus PPAR*α*-null mice treated with normal food in GSE5475; Study 2: Intestine-WY (KO/WT) is the study of mouse small intestine of wild type versus PPAR*α*-null mice treated with PPAR*α* agonist WY14643 for 5 days also in GSE5475; Study 3: Liver-CT (KO/WT) is the study of mouse liver of wild type versus PPAR*α*-null mice treated with normal food in GSE8295; Study 4: Liver-WY (KO/WT) is the study of mouse liver of wild type versus PPAR*α*-null mice treated with the PPAR*α* agonist WY14643 for 5 days also in GSE8295. On the other hand, additional 4 studies were followed to assess the effects of PPAR*α*-independent side. Study 5: Intestine-WT (CT/WY) is the study of wild type mice intestine with or without WY14643 treatment in GSE5475; Study 6: Intestine-KO (CT/WY) is the study of PPAR*α*-null mice intestine with or without WY14643 treatment in GSE5475; Study 7: Liver-WT (CT/WY) is the study of wild type mice liver with or without WY14643 treatment in GSE8295; Study 8: Liver-KO (CT/WY) is the study of PPAR*α*-null mice liver with or without WY14643 treatment in GSE8295. According to the eight comparisons above, we could identify dependent or independent pathways for PPAR*α* or WY14643. From study 1 and 3, we could get PPAR*α*-dependent pathway; from 2 and 4, we could get WY14643 PPAR*α*-dependent pathways; from study 5 and 7, WY14643-dependent pathways could be identified; from study 6 and 8, PPAR*α*-independent pathways could be identified.

For the assessment of the influence of preprocessing on the comparison, the data preprocessing was performed using software packages developed in version 2.6.0 of Bioconductor and R version 2.10.1 [25]. Each Affymetrix dataset was background adjusted, normalized and log2 probe-set intensities calculated using the robust multichip averaging (RMA) algorithm in Affy package [26].

### 2.2. Gene Set Enrichment Analysis

Here, we performed our gene set enrichment analysis on each study above to identify significantly related pathways and genes to either PPAR*α* or WY14643-dependent or independent effects by using Category package in version 2.6.0 of Bioconductor [27]. In our research, the gene sets represented by less than 10 genes were excluded. The *t*-statistic mean of the genes was computed in each pathway. Using a permutation test with 1000 times, the significantly changed pathways were identified with *P*  value ≤0.01. Accordingly, the significant pathways and genes between PPAR*α* knockout and wild type were then identified in either control or WY14643 (0.1% w/w) condition. Subsequently, the comparison of GSEA results based on the datasets between in small intestine and in liver of mice was performed to indicate the regulatory mechanisms of gene expression by PPAR*α* activation of each other.

## 3. Results and Discussion

Here, we used standardized microarray preprocessing and GSEA with comprehensive expression profiles in order to find greater data convergence and provide a systematic insight into the pathways altered between mouse hepatic and intestinal expression under the treatment of PPAR*α* knockout and WY14643 mixed diet or not.

### 3.1. Pathway Analysis of PPAR*α*-dependent Gene Regulation

**Figure 1:**
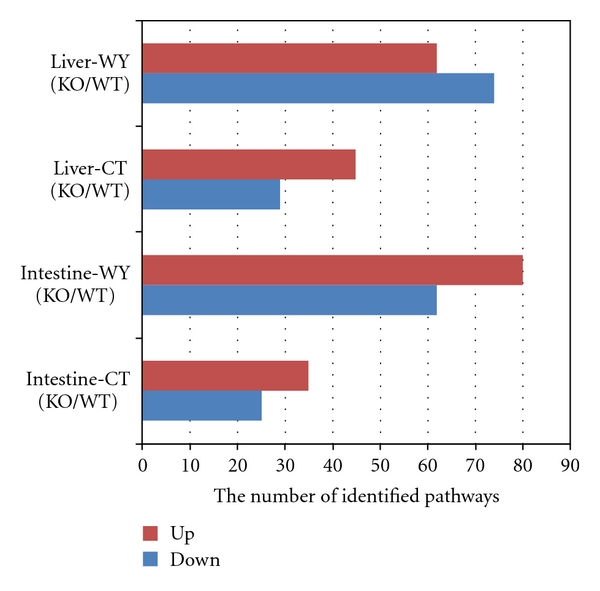
The number of identified up-or downregulated pathways in four studies of PPAR*α*-dependent side. The bar chart showing the number of significantly identified pathways (*P* ≤ 0.01) in our four studies of PPAR*α*-dependent side according to the data of Additional File 1 which is available online at doi:10:1155/2011/629728.*X*-axis represents the number of significantly identified pathways; *Y*-axis represents our four studies. The studies of Intestine-CT (KO/WT) and Liver-CT (KO/WT) are for identifying PPAR*α*-dependent pathways; the studies of Intestine-WY (KO/WT) and Liver-WY (KO/WT) are for identifying WY14643 PPAR*α*-dependent pathways, respectively, in mice small intestine and liver. Red color (up) is for upregulated pathways and blue color (down) is for downregulated pathways. There were 35 and 25 in study of Intestine-CT (KO/WT), the study in mouse small intestine of wild type versus PPAR*α*-null mice treated with normal food in GSE5475; 80 and 62 in study of Intestine-WY (KO/WT), the study in mouse small intestine of wild type versus PPAR*α*-null mice treated with PPAR*α* agonist WY14643 for 5 days; 45 and 29 in study of Liver-CT (KO/WT), the study in mouse liver of wild type versus PPAR*α*-null mice treated with normal food; and 62 and 74 in study of Liver-WY (KO/WT), the study in mouse liver of wild type versus PPAR*α*-null mice treated with the PPAR*α* agonist WY14643 for 5 days, respectively, for up-and down-regulated pathways.

Firstly, the common GSEA method was applied to the first 4 studies of PPAR*α*-dependent side. For individual analysis, we obtained the significant pathways in each dataset, which were summarized in Figure 1 and Additional File 1. The studies of Intestine-CT (KO/WT) and Liver-CT (KO/WT) are for identifying PPAR*α*-dependent pathways by comparison made between PPAR*α*-null (KO) and wild type (WT) mice without the treatment of WY14643; the studies of Intestine-WY (KO/WT) and Liver-WY (KO/WT) are for identifying WY14643 PPAR*α*-dependent pathways by comparison made between PPAR*α*-null (KO) and wild type (WT) mice with the treatment of WY14643, respectively, in small intestine and liver. As a result, There were 35 and 25 in study of Intestine-CT (KO/WT); 80 and 62 in study of Intestine-WY (KO/WT); 45 and 29 in study of Liver-CT (KO/WT); And 62 and 74 in study of Liver-WY (KO/WT), respectively, for up-and downregulated significantly identified pathways based on the permutation *P*-values (*P* ≤ 0.01). According to the bar chart in red color showing the number of significantly upregulated pathways, there was an obviously larger change from study 1 to study 2 (from 35 to 80 involved pathways) than from study 3 to study 4 (from 45 to 62 involved pathways). It suggested that the regulatory mechanism of PPAR*α* activation by WY14643 in mouse small intestine would be more complicated than in mouse liver, since more changed pathways were involved in mice intestinal PPAR*α* regulation.

**Figure 2:**
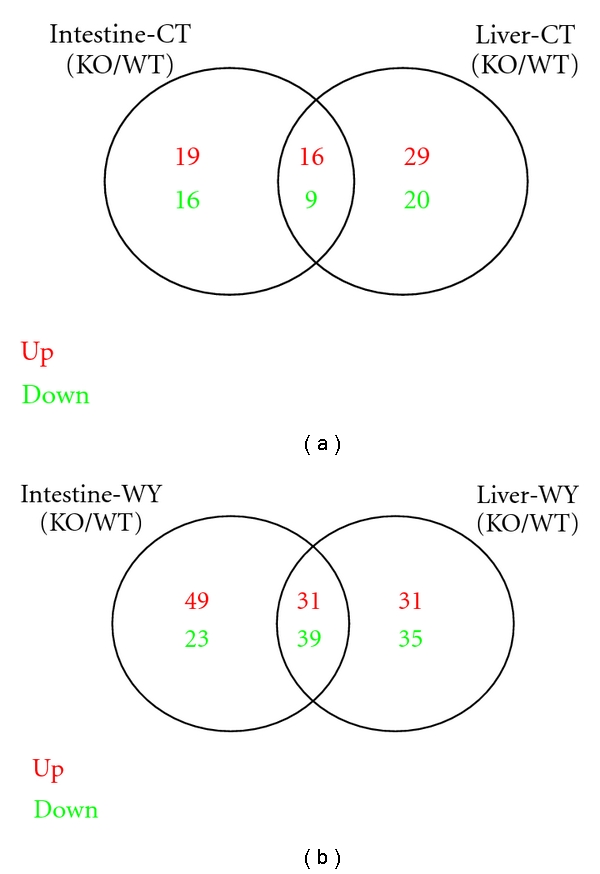
Comparison of PPAR*α*-dependent regulated pathways between in small intestine and liver of mice. Venn diagram showing the overlapping significantly identified pathways by the comparison of PPAR*α*-dependent regulated pathways between in small intestine and liver of mice. The studies of Intestine-CT (KO/WT) and Liver-CT (KO/WT) are for identifying PPAR*α*-dependent pathways; the studies of Intestine-WY (KO/WT) and Liver-WY (KO/WT) are for identifying WY14643 PPAR*α*-dependent pathways, respectively, in mice small intestine and liver. Pathways of *P*-value less than 0.01 were considered to be significantly regulated. Red color is for upregulated pathways and green color is for downregulated pathways. In Figure 2(a), there were 16 upregulated and 9 downregulated PPAR*α*-dependent pathways in common; in Figure 2(b), there were 31 upregulated and 39 downregulated WY14643 PPAR*α*-dependent pathways in common in both tissues of mice.

We further compared the GSEA results in each study. The overlapping pathways among each study from our comparative analysis were shown in Additional File 2. We then postulated that the pathways that appear consistently as significant in multiple studies are more likely to be important in PPAR*α* activation. For tissue effect, there were two comparisons that were of interest. Both of them are the comparisons of PPAR*α*-dependent regulated pathways between in small intestine and liver of mice. The overlaps were shown in Figure 2 and Additional File 2. Obviously, in the first comparison (Figure 2(a)) between study 1 and 3, there were 16 upregulated and 9 downregulated pathways which were PPAR*α*-dependent in both liver and intestine. In the second comparison (Figure 2(b)) between study 2 and 4, there were 31 upregulated and 39 downregulated WY14643 PPAR*α*-dependent pathways in common in both tissues of mice, which shared larger number than the first comparison result. It suggests that PPAR*α* ligand plays an important role in directing regulation of gene expression by PPAR*α*. Furthermore, in order to identify the highly common pathways appearing in both intestinal and hepatic studies, we compared the 25 common pathways in the first comparison with the 70 common pathways in the second comparison. As a result, there were 12 highly common pathways PPAR*α*-dependent in both tissues, including 4 upregulated and 8 downregulated pathways in PPAR*α* KO mice (see Table 3). The most downregulated pathways were metabolism related pathway, such as fatty acid metabolism, tryptophan metabolism, pyruvate metabolism, pantothenate and CoA biosynthesis, biosynthesis of unsaturated fatty acids, beta-Alanine metabolism, and glycerolipid metabolism identified in both liver and intestine [28–31]. Besides, PPAR signaling pathway was also identified as one of the most common downregulated pathways, which is part of endocrine system. Among the upregulated list, 3 pathways including lysosome, endocytosis and gap junction are related to cellular processes, which may lead to an enrichment of cholesterol in the plasma membrane [32]. The last one pathway named other glycan degradation is one member of glycan biosynthesis and metabolism. It suggested that the biosynthesis and metabolism of glycan may be downregulated with regard to PPAR*α* regulation in both liver and intestine of mice.

In addition, there were several tissue-specially identified pathways. For example, some signaling pathways such as MAPK signaling pathway, TGF-beta signaling pathway, VEGF signaling pathway, insulin signaling pathway, and some disease-related pathways such as type I diabetes mellitus and autoimmune thyroid disease were only identified in hepatic study. Interestingly, MAPK signaling pathway was PPAR*α*-dependent in liver but WY14643 PPAR*α*-dependent in small intestine, which was presented in brown color in Additional File 1. The relationships between PPAR*α* activation and some of these identified pathways in the tissue of liver were reported by previous studies [33–35]. By contrast, other some signaling pathways such as mTOR signaling pathway, Notch signaling pathway, T cell receptor signaling pathway were only identified in small intestinal study. Moreover, there were lots of cancer pathways identified both in small intestine and in liver, including endometrial cancer, prostate cancer, bladder cancer, and nonsmall cell lung cancer. But there were more cancer-related pathways identified in mice without WY14643 treatment, especially in liver, including pancreatic cancer and small cell lung cancer [36–38]. The activated PPAR*α* was known as a major regulator of hepatic miRNA expression and PPAR*α*-null mice were reported to be resistant to all of the pleiotropic effects of peroxisome proliferators, including cell proliferation and hepatocarcinogenesis [39, 40]. With regard to cancer pathways identified in the intestine, PPAR*α* was also thought to play an important role in prevention of intestinal cancer development.

### 3.2. Pathway Analysis of PPAR*α*-independent or WY14643-dependent Gene Regulation

**Figure 3:**
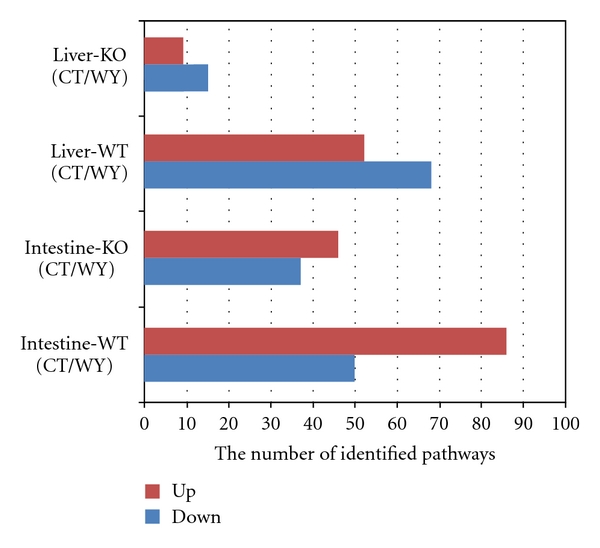
The number of identified up-or downregulated pathways in other four studies of PPAR*α*-independent side or WY14643-dependent side. The bar chart showing the number of significantly identified pathways (*P* ≤ 0.01)  in additional four studies of PPAR*α*-independent side or WY14643-dependent side according to the data of Additional File 1. *X*-axis represents the number of significantly identified pathways; *Y*-axis represents our four studies. The studies of Intestine-WT (CT/WY) and Liver-WT (CT/WY) are for identifying WY14643-dependent pathways; the studies of Intestine-KO (CT/WY) and Liver-KO (CT/WY) are for identifying PPAR*α*-independent pathways, respectively, in mice small intestine and liver. Red color (up) is for upregulated pathways and blue color (down) is for downregulated pathways. There were 86 and 50 in study of Intestine-WT (CT/WY), the study in mouse wild type intestine of comparison between with normal food and with the treatment of WY14643 in GSE5475; 46 and 37 in study of Intestine-KO (CT/WY), the study in mouse PPAR*α*-null intestine of the comparison between with normal food and with the treatment of WY14643 also in GSE5475; 52 and 68 in study of Liver-WT (CT/WY), the study in mouse wild type liver of comparison between with normal food and with the treatment of WY14643 in GSE8295; And 9 and 15 in study of Liver-KO (CT/WY), the study in mouse PPAR*α*-null liver of the comparison between with normal food and with the treatment of WY14643 also in GSE8295, respectively, for up-and downregulated pathways.

Subsequently, we also applied the similar GSEA approach to the other 4 studies of PPAR*α*-independent or WY14643-dependent side. The studies of Intestine-WT (CT/WY) and Liver-WT (CT/WY) are for identifying WY14643-dependent pathways by the comparison made between with and without WY14643 treatment in small intestine or liver of wide-type mice; the studies of Intestine-KO (CT/WY) and Liver-KO (CT/WY) are for identifying PPAR*α*-independent pathways by the comparison made between with and without WY14643 treatment in small intestine or liver of PPAR*α*-null mice. As a result, there were 86 and 50 in study of Intestine-WT (CT/WY); 46 and 37 in study of Intestine-KO (CT/WY); 52 and 68 in study of Liver-WT (CT/WY); and 9 and 15 in study of Liver-KO (CT/WY), respectively, for significantly up- and downregulated pathways (*P* ≤ 0.01). The details of identified pathways in the subsequent four studies were also shown in Additional File 1 and Figure 3. Obviously, there were less significant pathways identified in the study of Liver-KO (CT/WY) than in other studies. It suggested that the liver PPAR*α*-independent pathways are more sensitive to regulation by WY14643 while the intestine PPAR*α* pathways are more dependent on PPAR*α* activation by WY14643.

**Figure 4:**
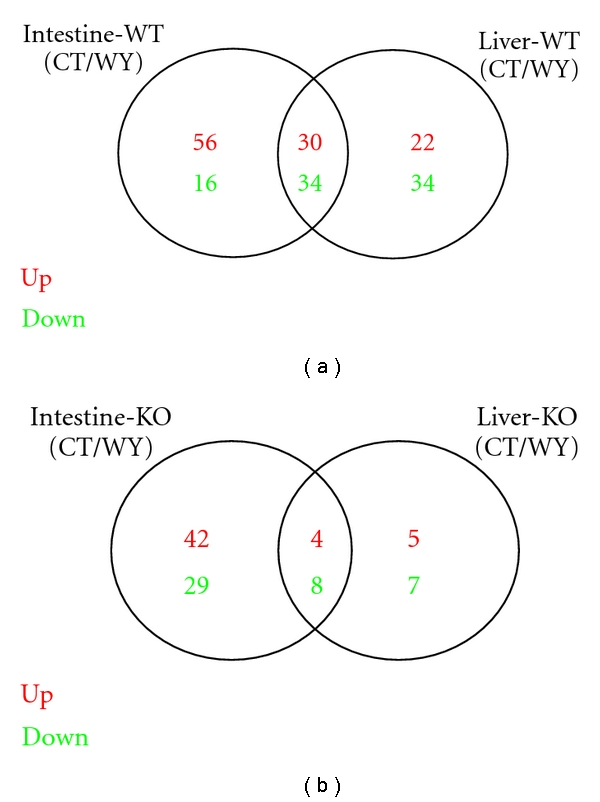
Comparison of PPAR*α*-independent or WY14643-dependent regulated pathways between in small intestine and liver of mice. Venn diagram showing the overlapping significantly identified pathways by the comparison of PPAR*α*-independent or WY14643-dependent regulated pathways between in small intestine and liver of mice. The studies of Intestine-WT (CT/WY) and Liver-WT (CT/WY) are for identifying WY14643-dependent pathways; the studies of Intestine-KO (CT/WY) and Liver-KO (CT/WY) are for identifying PPAR*α*-independent pathways, respectively, in mice small intestine and liver. Pathways of *P* value less than 0.01 were considered to be significantly regulated. Red color is for upregulated pathways and green color is for downregulated pathways. In (a), there were 30 upregulated and 34 downregulated WY14643-dependent pathways in common; in (b), there were only 4 upregulated and 8 downregulated PPAR*α*-independent pathways in common in both tissues of mice.

Further comparisons were performed to determine the common pathways of PPAR*α*-independent or WY14643-dependent effect based on the results of additional four PPAR*α*-independent or WY14643-dependent studies. The overlaps of significantly identified pathways by the comparison of PPAR*α*-independent or WY14643-dependent regulated pathways between in small intestine and liver of mice were exhibited in Figure 4 and the details were shown in Additional File 3. As a result, there were 30 upregulated and 34 downregulated WY14643-dependent pathways in common between study of 5 and 7 (Figure 4(a)); And there were only 4 upregulated and 8 downregulated PPAR*α*-independent pathways in common between study of 6 and 8 (Figure 4(b)). Furthermore, we compared the 64 common pathways in the first comparison with the 12 common pathways in the second comparison in order to identify the highly common pathways of PPAR*α*-independent appearing in both intestinal and hepatic studies. Interestingly, 4 highly common PPAR*α*-independent pathways were identified, including only 1 upregulated and 3 downregulated pathways by the comparison between with normal food and with the treatment of WY14643 (Table 3). All of the downregulated pathways were metabolism related, including gluconeogenesis, fatty acid metabolism and propanoate metabolism. Among them, the pathway of fatty acid metabolism was also identified in PPAR*α*-dependent studies, which had been confirmed to be related to the regulation of PPAR*α*. The only one upregulated pathway was systemic lupus erythematosus (SLE), one part of human diseases, which was reported as a heterogeneous disease involving several immune cell types and proinflammatory signals and the recent study of a novel mechanism through which PPAR*γ* regulated the inflammatory signal initiated by activation of CD40 was importantly implicated for the understanding of immunological mechanisms underlying SLE and the development of new treatment strategies [41].

There were other more tissue-special pathways identified in the studies of PPAR*α*-independent or WY14643-dependent effect. Although there were less significant pathways identified as PPAR*α*-independent in study of Liver-KO (CT/WY), the metabolism related pathway of keratan sulfate biosynthesis, the pathway of regulation of actin cytoskeleton, the pathways of prostate cancer and small cell lung cancer were not identified as hepatic PPAR*α*-independent but as WY14643-dependent ones in study of Intestine-WT (CT/WY). The findings were shown in blue color in Additional File 1. The pathway of keratan sulfate biosynthesis also named as glycosaminoglycan biosynthesis-keratan sulfate is one part of glycan biosynthesis and metabolism. Although there were few reports on the relationship between this pathway and PPARs, the effect of PPAR ligands modulating glucose metabolism was reported to alter the incorporation of metabolic precursors into proteoglycans synthesized by human vascular smooth muscle cells [42]. The pathway of regulation of actin cytoskeleton is cellular processes related. A previous study indicated that stimulation of PPAR*α* would enhance cardiomyogenesis in ES cells using a pathway that involves ROS and NADPH oxidase activity [43]. The pathways of prostate cancer and small cell lung cancer are both human diseases related. A study of the mechanism of DEHP tumorigenesis suggested that increases in oxidative stress induced by DEHP exposure may lead to the induction of inflammation and/or the expression of protooncogenes, resulting in a high incidence of tumorigenesis in PPAR*α*-null mice [44].

Finally, we compared our reanalysis results with existing analysis previously reported. According to genome-wide analysis of PPAR*α* activation in murine small intestine based on GSE5475, PPAR*α* was reported to be involved in the regulation of processes including immune system, cell proliferation and differentiation, and programmed cell death in intestine. It also remarkably indicated that almost all increased gene sets by PPAR*α* activation corresponded to metabolic processes, including fatty acids catabolism, mitochondrial oxidative metabolism, and several pathways that feed intermediately metabolites into these processes in small intestine of mice [14]. Based on comprehensive analysis of PPAR*α*-dependent regulation of hepatic lipid metabolism by expression profiling of GSE8295, many Gene Ontology classes including fatty acid beta-oxidation, acyl-CoA metabolism, leukotriene metabolism, and peroxisome organization and biogenesis were found to be governed by PPAR*α* [15]. Moreover, there were many cancer pathways identified both in small intestine and in liver, including endometrial cancer, prostate cancer, bladder cancer, and nonsmall cell lung cancer according to our comparative study of mouse hepatic and intestinal gene expression profiles under PPAR*α* knockout by gene set enrichment analysis. According to transcriptome analysis of endometrial cancer, peroxisome proliferator-activated receptors were reported as potential therapeutic targets in endometrial cancer [45]. The overexpression of PPAR*α* in advanced prostate cancer may indicate a role in tumor progression with the potential involvement of dietary factors through fatty acid and steroid hormone signaling pathways [46]. PPAR*α* and PPAR*γ* were reported to be coexpressed, functional and show positive interactions in the rat urinary bladder urothelium and oxidative stress may contribute to rat urothelial carcinogenesis by dual-acting PPAR*α* and PPAR*γ* agonists [47]. The result of a previous study suggested that the enhancement of PPAR*γ* activity with its ligands, and the suppression of PPAR*α* with its inhibitors, may prevent the formation of lung tumors, as well as accelerate the therapy of lung cancer [48]. By our further comparisons, we also provided more tissue-special pathways PPAR*α*-dependent or independent. We have also identified 12 PPAR*α*-dependent and 4 PPAR*α*-independent highly common in both small intestine and liver of mice. Some of them have been approved by previous studies, such as fatty acid metabolism of both PPAR*α*-dependent and independent effect and PPAR signaling pathway of PPAR*α*-dependent effect. While we have identified some unique PPAR-dependent or independent pathways no previously identified, including the PPAR-dependent pathway of other glycan degradation associated with glycan biosynthesis and metabolism and the PPAR-independent pathway of systemic lupus erythematosus related to immune system diseases.

### 3.3. Tissue-Specific Genes in the Highly Common PPAR*α*-Dependent Pathways

Our analysis revealed that there were 12 highly common PPAR*α*-dependent pathways identified in both tissues, including 4 upregulated and 8 downregulated pathways. In order to identify tissue-specific PPAR*α* regulated genes, we further analyzed the datasets and listed the candidate genes of these highly common pathways in each study (see in Additional File 3). Obviously, the number of the candidate genes of each pathway in hepatic studies was always more than the number in small intestinal studies, which indicates that more regulated genes participate in PPAR*α* activation in murine liver. On the other hand, the genes in red color in Additional File 3 were our candidate ones of liver-specific genes in each highly common pathway, which just appeared in hepatic studies (Study 3 and 4). The genes of *Acsl3* and *Cyp4a29* were identified in the pathway of fatty acid metabolism just in hepatic studies [49, 50]. Other liver-specific genes in other pathways were as follows: *Ccbl1, Maob,* and *Tph2* were identified in the pathway of tryptophan metabolism; *Pank2* and *Pank4* were identified in pathway of pantothenate and CoA biosynthesis; *Yod1* was in pathway of biosynthesis of unsaturated fatty acids; *Cpt1c* and *Cyp4a29* were in PPAR signaling pathway; *Cndp1* was in pathway of beta-Alanine metabolism; *Agpat2, Agpat4, Dgki, Dgkb,* and *Dgkh* were in the pathway of glycerolipid metabolism; *Aga* and *D230014K01Rik* were in the pathway of other glycan degradation; *Npc1, Arsb, Ctso, Aga, Gga3,* and *Ap4e1* were in the pathway of lysosome; *Adcy5, Tubb1, Adcy1, Drd1a, Grm5, *and *Gucy1a2* were in the pathway of gap junction. Moreover, the pathway of endocytosis was a unique one, in which many genes were identified. For example, *Psd2, Smurf1 *and *Arfgap3 *were just identified in study 3 and 4. We suggest that all of the identified genes could be seen as our candidate liver-specific genes, but needed of further study.

## 4. Conclusions

Our study strongly indicates that the consolidation of the two different tissue (liver and small intestine) expression data sets can increase data quality and can lead to biologically more meaningful results. We have performed 8 PPAR*α* or WY14643-dependent and independent studies by using GSEA on microarray datasets and making comparisons. In intestinal studies, we have identified 35 and 25 PPAR*α*-dependent, 80 and 62 WY14643 PPAR*α*-dependent, 86 and 50 WY14643-dependent and 46 and 37 PPAR*α*-independent up-and downregulated pathways. In hepatic studies, we have identified 45 and 29 PPAR*α*-dependent, 62 and 74 WY14643 PPAR*α*-dependent, 52 and 68 WY14643-dependent and 9 and 15 PPAR*α*-independent up-and downregulated pathways. We suggested that the regulatory mechanism of PPAR*α* activation by WY14643 in mouse small intestine would be more complicated than in mouse liver since more changed pathways were involved in mice intestinal PPAR*α* regulation. Moreover, there were several cancer-related pathways such as pancreatic cancer and small cell lung cancer identified in mice without WY14643 treatment especially in liver, which indicated that PPAR*α* was thought to play an important role in prevention of cancer development. Finally, we found 12 PPAR*α*-dependent pathways and 4 PPAR*α*-dependent pathways identified highly common in both tissues of mice. Most of them were metabolism related, such as fatty acid metabolism, tryptophan metabolism, pyruvate metabolism with regard to PPAR*α* regulation and gluconeogenesis, and propanoate metabolism independent of PPAR*α* regulation. Furthermore, some pathways such as keratan sulfate biosynthesis, the pathway of regulation of actin cytoskeleton, the pathways associated with prostate cancer and small cell lung cancer were not identified as hepatic PPAR*α*-independent but as WY14643-dependent ones in intestinal study. We have identified some unique PPAR-dependent or independent pathways no previously identified, including other glycan degradation and systemic lupus erythematosus. We also provided some novel hepatic tissue-specific marker genes.

## Supplementary Material

Additional file 1 Significantly identified pathways in each study.
Additional file 2 The overlapped pathways in each comparison of PPAR*α* dependent studies.
Additional file 3 The overlapped pathways in each comparison of PPAR*α* independent studies.
Additional file 4 Candidate genes for highly common down- and up-regulated pathways in each study.

## Figures and Tables

**Table 1:** Summary of re-analyzed datasets.

Dataset	Period	Platform	Probes	Tissue	No. of
					samples
GSE5475	Aug, 2006	MOE430A	22k	Intestine	12
GSE8295	Jun, 2007	Mouse430_2	45k	Liver	16

There were two datasets named GSE5475 and GSE8295 for our reanalysis studies, which were both contributed by Guido Hooiveld in different periods (August in 2006 and June in 2007), respectively, for mouse small intestine and liver. The platforms used here were Affymetrix 430A arrays and Affymetrix mouse genome 430 2.0 GeneChip arrays. The sample sizes were 12 and 16 respectively.

**Table 2:** Summary of 8 comparison studies.

Study	Comparison name	Identified pathways
1	Intestine-CT (KO/WT)	PPAR*α*-dependent pathway in intestine
3	Liver-CT (KO/WT)	PPAR*α*-dependent pathway in liver
2	Intestine-WY (KO/WT)	WY14643 PPAR*α*-dependent pathways in intestine
4	Liver-WY (KO/WT)	WY14643 PPAR*α*-dependent pathways in liver
5	Intestine-WT (CT/WY)	WY14643-dependent pathways in intestine
7	Liver-WT (CT/WY)	WY14643-dependent pathways in liver
6	Intestine-KO (CT/WY)	PPAR*α*-independent pathways in intestine
8	Liver-KO (CT/WY)	PPAR*α*-independent pathways in liver

There were 8 comparison studies related to PPAR*α* in mice intestine or in liver included in our research. Each of the 8 comparisons identified dependent or independent pathways for PPAR*α* or WY14643.

CT: mice were treated with normal food (control).

WY: mice were treated with the synthetic PPAR*α* ligand WY14643 (0.1% w/w) mixed in the food.

KO: PPAR*α*-null (129S4/SvJae) mice.

WT: wild type (129S1/SvImJ) mice.

**Table 3:** The highly common down and up regulated pathways identified in studies of PPAR*α*-dependent or independent effects both in liver and small intestine tissues of mice.

Pathways	PPAR*α*-dependent (KO/WT)	PPAR*α*-independent (CT/WY)
Upregulated	00511: other glycan degradation	05322: systemic lupus erythematosus
	04142: lysosome	
	04144: endocytosis	
	04540: gap junction	

Downregulated	00071: fatty acid metabolism	00010: glycolysis/Gluconeogenesis
	00380: tryptophan metabolism	00071: fatty acid metabolism
	00620: pyruvate metabolism	00640: propanoate metabolism
	00770: pantothenate and CoA biosynthesis	
	01040: biosynthesis of unsaturated fatty acids	
	03320: PPAR signaling pathway	
	00410: beta-Alanine metabolism	
	00561: glycerolipid metabolism	

There were 12 highly common pathways in comparisons of PPAR*α*-dependent studies, including 4 upregulated and 8 downregulated pathways in both liver and small intestine of mice. The PPAR*α*-dependent comparisons were made between PPAR*α* null and wild type mice; By contrast, there were only 4 highly common pathways in comparisons of PPAR*α*-independent studies, including 1 upregulated and 3 downregulated pathways in both liver and small intestine of mice. The comparisons were made between with normal food and with WY14643 treatment.
